# Mass Spectrometry-Based Proteomic Study Makes High-Density Lipoprotein a Biomarker for Atherosclerotic Vascular Disease

**DOI:** 10.1155/2015/164846

**Published:** 2015-05-18

**Authors:** Chiz-Tzung Chang, Chao-Yuh Yang, Fuu-Jen Tsai, Shih-Yi Lin, Chao-Jung Chen

**Affiliations:** ^1^College of Medicine, China Medical University, 91 Hsueh-Shih Road, Taichung 40402, Taiwan; ^2^Division of Nephrology, China Medical University Hospital, 2 Yu-Der Road, Taichung 40447, Taiwan; ^3^L5 Research Center, China Medical University Hospital, Taichung, Taiwan; ^4^Section of Cardiovascular Research, Department of Medicine, Baylor College of Medicine, One Baylor Plaza, Houston, TX 77030, USA; ^5^Department of Medical Genetics, Pediatrics and Medical Research, China Medical University Hospital, Taichung 40447, Taiwan; ^6^Proteomics Core Laboratory, Department of Medical Research, China Medical University Hospital, Taichung 40447, Taiwan; ^7^Graduate Institute of Integrated Medicine, China Medical University, Taichung 40402, Taiwan

## Abstract

High-density lipoprotein (HDL) is a lipid and protein complex that consists of apolipoproteins and lower level HDL-associated enzymes. HDL dysfunction is a factor in atherosclerosis and decreases patient survival. Mass spectrometry- (MS-) based proteomics provides a high throughput approach for analyzing the composition and modifications of complex HDL proteins in diseases. HDL can be separated according to size, surface charge, electronegativity, or apoprotein composition. MS-based proteomics on subfractionated HDL then allows investigation of lipoprotein roles in diseases. Herein, we review recent developments in MS-based quantitative proteomic techniques, HDL proteomics and lipoprotein modifications in diseases, and HDL subfractionation studies. We also discuss future directions and perspectives in MS-based proteomics on HDL.

## 1. Introduction

High-density lipoprotein (HDL) is a heterogeneous complex of differing size, density, surface charge, and lipoprotein content [1]. Serum HDL level is thought to be inversely related with atherosclerotic vascular disease (ASVD) risk [2, 3]. HDL can protect against atherosclerosis via its cholesterol acceptor and effects in antioxidation, anti-inflammation, and antiapoptosis [4–6]. However, several clinical studies using therapeutic serum HDL-elevating agents failed to demonstrate their clinical benefits [7]. Recent studies have shown that HDL protein oxidation, glycation, carbamylation, and other modifications can compromise HDL function and result in increased ASVD risk [8–10]. Thus, protein composition changes or modifications on HDL can act as biomarkers for ASVD. With the improvement in subfractionation of HDL complexes and the advance in MS-based proteomic approaches, it is feasible to analyze HDL proteome and their modifications, giving a global view of biological processes and molecular functions of HDL proteins in diseases.

## 2. HDL Composition and Protein Heterogeneity

HDL (density = 1.063–1.210 g/mL) [11] is composed of approximately equal mass portions of proteins and lipids, whose molar differences result in HDL heterogeneity [12]. Indeed, HDL particles carry more than 80 different types of proteins and 100 types of lipid species [13, 14]. In terms of structure, typically, HDL is broadly spherical (diameter 70–100 Å), but nascent HDL is discoidal [15].

**Figure 1:**
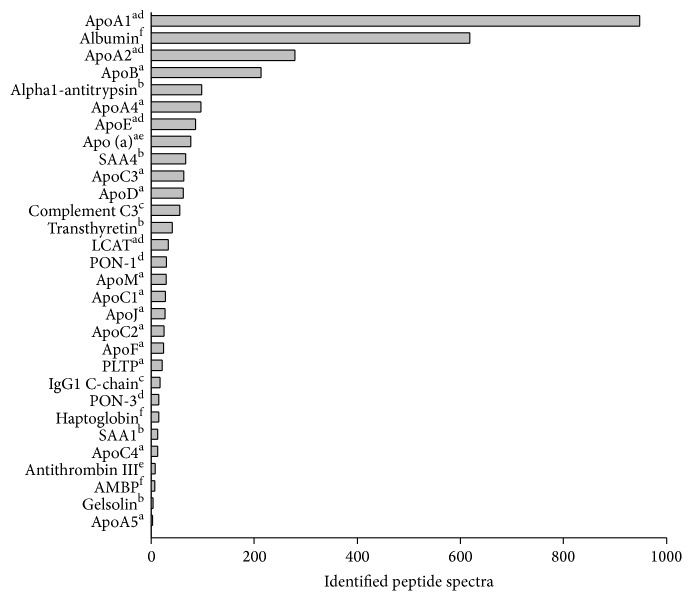
HDL proteome analysis by nano-LC-MS/MS. The identified peptide spectra of major proteins were presented as horizontal bars and their main functions were categorized. a: lipid transport protein; b: acute phase protein; c: complement and immunological pathway protein; d: antioxidant protein; e: hemostasis-associated protein; f: other cellular processes.

Nanoflow liquid chromatography (nano-LC) coupled online with nanoelectrospray ionization-tandem MS (nano-ESI-MS/MS) has become the gold standard for high throughput identification of proteins in complex biological samples.  Figure 1 showed the major identified proteins on HDL by nano-LC-MS/MS. These proteins can be categorized into lipid transporter proteins, complement pathway proteins, immunological pathway proteins, acute phase proteins, and antioxidant and hemostasis-associated proteins (Figure 1). HDL proteome diversity was compatible with multifunctional roles of HDL on lipid metabolism, oxidation, immune reaction, inflammation, and hemostasis. Apolipoprotein A1 (ApoA1) and ApoA2 take up 70% and 20% of HDL protein mass, respectively [16], but ApoA4, ApoE, ApoC, ApoJ, and others are also present in less amounts. ApoE is polymorphic and has three major isoforms—ApoE2, ApoE3, and ApoE4 [17]. ApoC is a family with four members, ApoC1, ApoC2, ApoC3, and ApoC4, each with several isoforms [18, 19]. HDL also contains small amounts of miscellaneous proteins, for example, acute-phase protein, HDL-associated enzymes, for example, paraoxonase-1 (PON-1), lecithin-cholesterol acyltransferase (LCAT), and lipoprotein-associated phospholipase A2 (Lp-PLA2). HDL lipids comprise a phosphatidylcholine bilayer and a cholesteryl ester core, including a small amount of cholesterol and triglyceride [20]. The cholesteryl ester and triglyceride content of HDL can affect HDL size; particles with higher triglyceride content tend to be larger [21]. Cholesteryl ester transfer proteins (CETP) mediate the transfer of cholesteryl esters from HDL to low-density lipoproteins (LDL) or very low-density lipoproteins (VLDL) in exchange for triglycerides. Similarly, phospholipid transfer proteins (PLTP) transfer phospholipids between HDL and VLDL. These two transfer proteins and other plasma enzymes, such as hepatic lipase and secretory phospholipase A2, play important roles in HDL composition regulation [2]. ApoC family members also play an important role in the metabolism and compositions of HDL. ApoC1 inhibits CETP; ApoC2 activates lipoprotein lipase; ApoC3 inhibits lipoprotein lipase [19, 22, 23]. Whilst ApoC4 has a lower plasma concentration than Apo C1, C2, or C3, it exists primarily in VLDL and HDL and plays a role in lipoprotein metabolism.

HDL composition can also be affected by inflammation [24] or disease such as uremia [25], psoriasis [26], or diabetes [27]. Patients with low HDL-cholesterol (HDL-C) expressed higher serum levels of C-reactive proteins [28]. In addition to proteins and lipids, HDL in both healthy and sick individuals also contains small amounts of microRNAs [29]. In fact, microRNAs have been shown to govern HDL metabolism and reverse cholesterol transport (RCT), the export of cholesterol from macrophages and peripheral tissues to the liver for biliary excretion [30].

## 3. HDL Functions and Associated Diseases

In the vascular wall, macrophages and resident cells engulf oxidized and other modified LDLs, leading to atherosclerosis [31]. Vasoprotective and antiatherogenic HDL can reverse the atherosclerotic process. The antiatherogenic function comes mainly from RCT [5]. ATP-binding cassette transporters ABCA1 and ABCG1 transfer cholesterol in macrophage foam cells to lipid-free ApoA1 and ApoE [32] or from macrophages to HDL particles [33], respectively. The ABCA1-ApoA1 axis plays a major role in cholesterol efflux [34]; therefore, RCT can predict atherosclerosis in humans [4]. Recently, RCT has also been reported to affect the macrophage immune response [35], nitric oxide (NO) production by endothelial nitric oxide synthase (eNOS), and insulin secretion from pancreatic islet cells [34, 36]. Through enhancement of endothelial NO formation and vasodilation, HDL inhibits endothelial cell apoptosis and stimulates endothelial cell repair and vasoprotection [37, 38]. However, some believe that HDL vasoprotection may be independent of the NO pathway [39, 40]. HDL also has antioxidant, antiplatelet, and anti-inflammatory effects, which are all individually antiatherogenic and vasoprotective in nature. Additionally, HDL metabolizes triglyceride-rich lipoprotein by donating ApoE or ApoC to nascent chylomicron or VLDL [31].

ApoA1 and HDL-associated enzymes play key roles in RCT, antiendothelial cell apoptosis [41], antioxidation, and anti-inflammation [9]. In an esterase with peroxidase-like activity [42], PON-1 associates with ApoA1 and exerts its atheroprotective effect by preventing LDL from oxidative modifying [43, 44]. Lp-PLA2 is not only a novel risk factor for atherosclerosis [45, 46], but also an antioxidant due to its role in reducing oxidized lipids [47]. LCAT plays an important role in RCT by esterifying cholesterol to facilitate HDL maturation and subsequent cholesterol excretion in the liver [5].

Low plasma HDL-C has long been known as a significant independent risk factor of atherosclerosis in patients with coronary artery disease (CAD), diabetes [48, 49], and uremia [20]; it persists even after adjustment for obesity and hypertriglyceridemia [50]. Meta-analysis from large prospective studies demonstrated a 2-3% decrease in CAD risk for every 1 mg/dL increase in HDL-C plasma levels [51]. Furthermore, the Framingham study revealed a significant CAD risk increase in patients with plasma HDL-C of lower than 34 mg/dL [3]. However, established medications targeted at elevating HDL-C, such as statin, nicotinic acid, or fibrate, fail to reduce atherosclerosis risk [52]. Novel agents such as CETP inhibitors focused on raising HDL-C, although promising, lack verification of their efficacy from large, long-term, randomized control trials [53]. Many other small molecules or peptides are still undergoing different stages of basic or clinical studies and have not yet been put into clinical use [54].

Recent studies have indicated that biochemical alterations or changes in the protein content of HDL may lead to HDL dysfunction in patients with CAD, ischemic stroke, Alzheimer's disease, or uremia [55, 56]. For example, patients with high atherosclerotic vascular disease risks, such as smokers, exhibit lower HDL PON-1 mass and activity than those with low risks, such as nonsmokers [57]; moreover, uremia patients have lower LCAT levels and activities than patients without uremia [58].

## 4. MS-Based Quantitative Proteomic Approaches

To compare protein expression between different biological samples, quantitative proteomics has developed both gel-based and gel-free methods. In two-dimensional polyacrylamide gel electrophoresis (2D-PAGE), thousands of proteins are separated in a gel matrix based on isoelectric point (first dimension) and molecular weight (second dimension). The introduction of fluorescent Cy dyes (Cy2, Cy3, and Cy5) in 2D-PAGE greatly improved protein quantitation precision [59].

**Figure 2:**
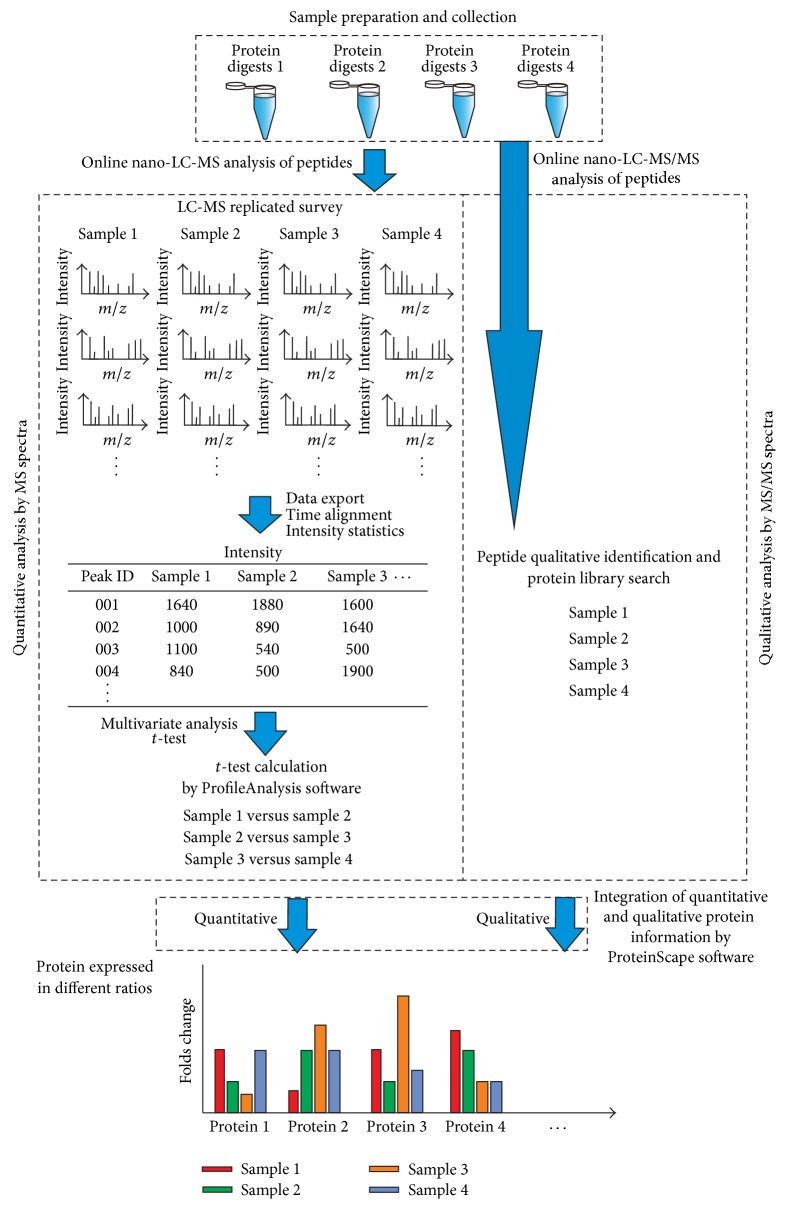
The flow chart of label-free quantitative proteomics based on extracted ion chromatography. The label-free quantitative proteomics was achieved by the software packages of DataAnalysis, ProfileAnalysis, and ProteinScape from Bruker Daltonics. Each sample group of digested proteins was tested by nano-LC-MS with replicated runs for quantitation of peptide ions. MS/MS spectra were also acquired by nano-LC-MS/MS analysis of these digested samples for protein identification. The intensity and elution time of each peptide ions were recorded as a quantitative “molecular feature.” These molecular feature ions acquired from different nano-LC-MS runs were aligned according to their accurate masses and reproducible LC retention time. Peptide peaks with expression ratios between two sample groups were calculated with *t*-test method in ProfileAnalysis. These*t*-test results were further transferred to ProteinScape and combined with their protein identification results for integrating both quantitative and qualitative information of each protein in all sample groups.

Gel-free quantitative proteomics divides into label-free and label-based methods. In label-free quantitative proteomics, spectral counting of a protein or ion intensity of each peptide can be used to calculate the relative protein expression level. The spectral count strategy is based on the positive correlation of protein abundance with sequence coverage and the number of identified MS/MS spectra [60]. Although spectral counting is efficient, cost effective, and easy to perform, the precision and the analysis of complex protein mixtures are questionable. The ion peak intensity method, which is based on the linear correlation between ion peak intensity and peptide concentration within the dynamic range of a mass analyzer [61], is a more reliable strategy; however, this method requires a package of reliable statistical software. One of the label-free approaches based on comparing peak intensity extracted from nano-LC-MS scan with *t*-test is shown in Figure 2. Each sample group of digested proteins has to be performed with replicated nano-LC-MS runs for quantitation of peptide ions and nano-LC-MS/MS runs for acquiring MS/MS spectra for protein identification.

**Figure 3:**
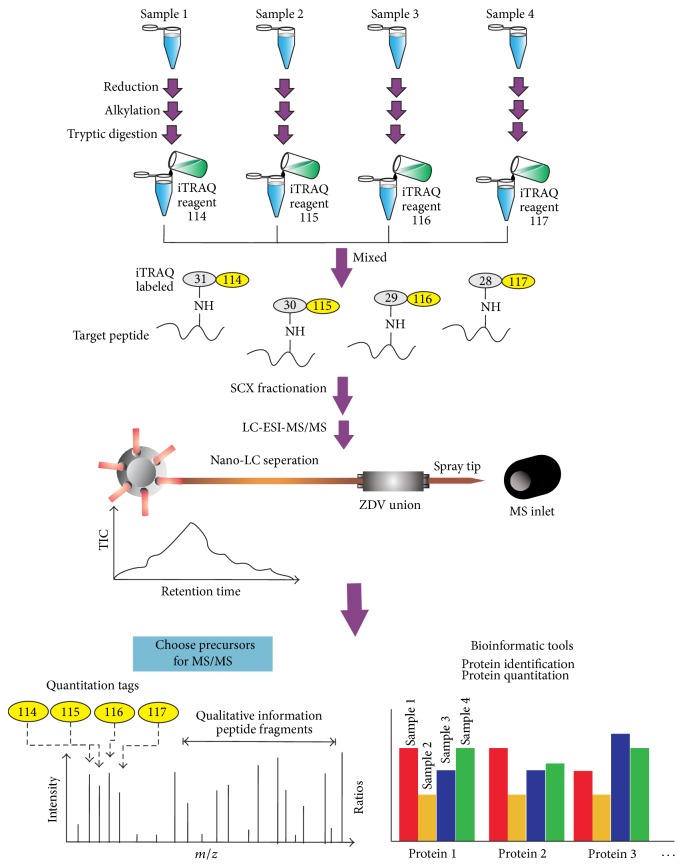
General flow chart of 4-plex iTRAQ labeling with SCX fractionation and nano-LC-MS/MS analysis. Proteins of each sample group were reduced and alkylated followed by enzymatic digestion. Four-plex iTRAQ reagents were used to label 4 protein digested samples. The combined mass of the reporter (114, 115, 116, and 117 Da) and the balance groups of labeling tag is 145 Da. After labeling, the 4 iTRAQ-labeled samples were mixed to become one sample followed by desalting purification. SCX fractionation is an optional method to reduce complexity of peptide mixtures prior to nano-LC-MS/MS analysis. The MS/MS spectra were searched against protein database for protein identification. Bioinformatics tools are used to integrate the protein identification and quantitation information with mass tags of 114, 115, 116, and 117 Da in their corresponding peptide MS/MS spectra.

Labeling methods include isotope coded protein labeling (ICPL), isobaric tags for relative and absolute quantification (iTRAQ), tandem mass tag (TMT), and stable isotope labeling with amino acids in cell culture (SILAC), among others. iTRAQ is currently the most popular and widely utilized labeling method in quantitative proteomics and utilizes an isobaric tag (total mass of 145 Da) consisting of a reporter group (mass = 114–117 Da), a balance group (mass = 31–28 Da), and a peptide reactive group. Upon peptide fragmentation, measurement of the reporter ion intensities (e.g., 114–117 Da of 4-plex iTRAQ kit) enables the relative quantitation of proteins in each sample [62]. To increase the numbers of identified proteins, strong cation exchange (SCX) chromatography is usually used to subfractionate iTRAQ-labeled peptides. The general analytical flow chart of labeling peptides with iTRAQ followed by SCX fractionation and nano-LC-MS/MS analysis is shown in Figure 3. However, iTRAQ quantitation precision may be interfered by the mixed MS/MS contribution occurring during precursor selection when analyzing highly complex mixtures [63].

Unlike other chemically labeled methods, SILAC is metabolically labeled in cell culture with heavy forms of amino acids. Because SILAC-labeled samples can be mixed immediately before any further processing steps, this minimizes quantitative errors due to sample handling. SILAC has been widely applied in mammalian cell culture and simple microorganisms and successfully extended to fruit flies and mice by feeding them lysine-labeled yeast and diet, respectively [64, 65]. Several SILAC-labeled cell lines can also be mixed together (super-SILAC) and serve as the spike-in standard SILAC for human tissue proteome study [66]. However, SILAC is hardly ever applied in HDL proteomics.

Although 2D-PAGE analysis is laborious, 2D-PAGE has been applied for observing oxidative damage of ApoA1 [67] and glycation [68] with high protein separation efficiency. Unlike labeling approaches, label-free approaches are still limited to their quantitative accuracy in the integrated data of two-dimensional separated subfractions. Due to the limited protein numbers on HDL, it is feasible to detect complete proteins on HDL in a single nano-LC-MS/MS run. However, once considering 4 or more sample groups for comparison, labeling methods were recommended as more time saving approaches.

In addition to nano-LC-MS/MS-based quantitative proteomics methods, matrix-assisted laser desorption/ionization (MALDI) has also been used as a relatively quantitative tool to rapidly discover biomarkers in bacteria [56], serum, urea, and saliva. With the use of stationary phases-coated magnetic particles or sample plates (e.g., surface-enhanced laser desorption/ionization (SELDI) [69]) for specific biomolecular purification, MALDI-time of flight (TOF) can be used to rapidly detect specific compounds.

**Figure 4:**
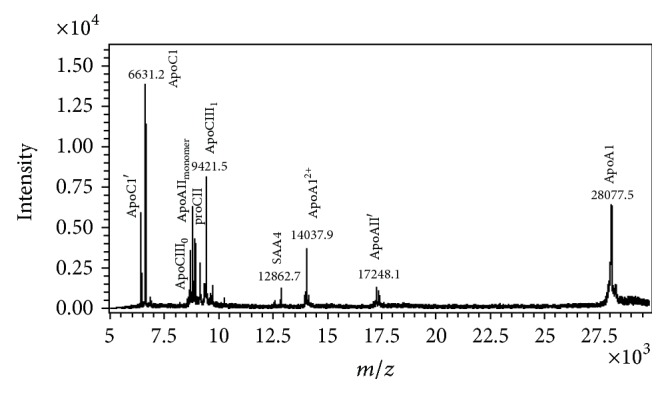
MALDI-TOF-MS analysis of human HDL. HDL was dialyzed against degassed 20 mM Tris-HCl, 0.5 mM EDTA, and 0.02% NaN_3_, pH 8.0 at 4°C with 3 buffer changes in 24 hours. MALDI-TOF-MS (Ultraflex III TOF/TOF, Bruker Daltonics, Germany) with linear mode was used to identify the major apolipoproteins and their isoforms in HDL. Other detailed experimental settings can be referred to in [16]. ApoC1 (calculated mass: 6630.6* m/z*); ApoC1′: ApoC1 minus N-terminus Thr-Pro (calculated mass: 6432.4* m/z*); ApoCIII_0_ (calculated mass: 8765.7* m/z*); ApoAII monomer: single chain ApoAII (calculated mass: 8809.9* m/z*); proCII (calculated mass: 8914.9* m/z*); SAA4 (calculated mass: 12863.2* m/z*); ApoAII′: apoAII minus C-terminus-Gln (calculated mass: 17253.7* m/z*); ApoAI (calculated mass: 28078* m/z*).

However, MALDI-TOF-based protein profiling is still constrained by poor sensitivity in detection of larger proteins (>30 kDa) and limited ion peaks in complex samples. Fortunately, because major lipoproteins on HDL are smaller than 30 kDa, MALDI-TOF is quite suitable for revealing expression changes of major HDL lipoproteins and their isoforms in different disease backgrounds (Figure 4). More comprehensive protein profiling can be observed after HDL subfractionation [16].

## 5. HDL Fractionation Techniques

The heterogeneity of HDL stems from its variation in density, size, composition, and surface charge [1]. Fractionating HDL into subgroups may facilitate the compositional and functional studies of HDL. In MS analysis, sample fractionation can reduce sample complexity, therefore decreasing ion suppression effects of ESI and MALDI to improve detection sensitivity. Most commonly, HDL is separated into subclasses using density gradient ultracentrifugation, which can be used to separate HDL2 (*d* = 1.063–1.125 g/mL) and HDL3 (*d* = 1.125–1.210 g/mL) [70]. Another frequently used method is subfractionation by size, which is accomplished by nondenaturing gradient gel electrophoresis or nuclear magnetic resonance (NMR) spectroscopy [71]. In order of decreasing size, HDL can be separated into HDL2b, HDL2a, HDL3a, HDL3b, and HDL3c [72]. HDL can also be separated based on surface charge into pre-beta, alpha, and pre-alpha HDL using agarose gel electrophoresis [73]. Some studies have classified HDL according to ApoA1/ApoA2 composition into LipA1, LipA1/A2, and LipA2 [74]. The above methods are limited in that they may lose of some material during ultracentrifugation, lack standardized methods for gel electrophoresis, and include unknown assumptions in the NMR data analysis software [75].

Recently, we successfully fractionated HDL from normal adults according to electronegativity. HDL can be separated into five subfractions (H1–H5) with increasing electronegativity using a fast protein LC anion-exchange column [16]. When subjected to SDS gel electrophoresis, apolipoprotein distributions in H1 to H5 differed. ApoC1, which carries a strong positive charge at physiological plasma pH, is located mainly in H1. On the other hand, ApoC3, which carries several negatively charged sialic acid residues, is found in H5. The amounts of ApoA1 decreased from H1 to H5. The same lipoprotein protein distribution in HDL subfractions was determined by MALDI-TOF-MS.

The distributions of major HDL-associated enzymes, such as PON-1, Lp-PLA2, and LCAT, also differ among these 5 subfractions. LCAT levels are higher in H4 and H5 than in H1–H3, and LCAT activity is in agreement with this distribution. The RCT function of HDL subfractions, however, is lowest in H5 due to its lowest ApoA1 content. It is apparent that subgrouping HDLs according to electronegativity can separate apolipoproteins with good resolution for HDL composition determination and functional studies. This novel HDL subgrouping method provides an additional scope to study HDL compositional changes and biofunctions in various diseases such as diabetes, hyperlipidemia, and uremia.

## 6. HDL Proteomics in Disease

HDL proteomics have been extensively reviewed [13, 76, 77]; therefore, in this review, we focus on recently published papers (Table 1). The proteome can also be altered after disease treatment. Jorge et al. reported that HDL proteome in CAD patients changed dynamically according to disease status. HDL proteins altered after percutaneous transluminal coronary angioplasty (PTCA). Several apolipoproteins and fibrinogen-like protein increased, but antithrombin III, annexin A1, and several immunoglobulins decreased after PTCA-induced atheroma plague rupture. Protective properties of HDL were impaired after PTCA [78].

Vaisar et al. used a label-free quantitative proteomic method (peptide index) to study differential protein expression in HDL3 of CAD patients and identified 48 different proteins in HDL and HDL3 fractions, which were categorized as lipid metabolism, proteinase inhibition, acute-phase response, and complement regulation by gene ontology (GO) analysis. PON-1, ApoC4, ApoA4, complement C3, and ApoE HDL3 levels in CAD patients were significantly increased compared to healthy controls. Because ApoE levels are reported to be lower in HDL2 from subjects with CAD, it is possible that redistribution of ApoE from HDL2 to HDL3 impairs cholesterol efflux and promotes formation of macrophage foam cells* in vivo *[14].

Recently, the same team used another proteomic profiling method with MALDI-TOF analysis of trypsin-digested proteins from HDL2. A partial least squares discriminant analysis (PLS-DA) model based on MALDI-MS signals (24 peptide signals) containing some peptides of ApoA1 (oxidation at Met112), ApoC3 (upregulated), lipoprotein(a) (upregulated), and ApoC1 (downregulated) accurately classified CAD and control subjects [8].

The HDL proteome in hemodialysis (HD) patients has been investigated by iTRAQ labeling, IEF peptide separation (OFFGEL Fractionator, Agilent), and nano-LC-MS/MS [79]. Of the 303 proteins identified, 122 were further selected using stringent criteria, and among them 40 displayed differential expression in HD patients compared to healthy groups. These differentially expressed proteins have been implicated in many functions including lipid metabolism, inflammatory response, the complement and coagulation cascade, and endopeptidase inhibitor activity. The increase of ApoC2/ApoC3 and decrease of serotransferrin in HDL of HD patients compared with healthy groups were identified and validated. Increased ApoC2 and ApoC3 imply abnormal transfer of ApoC to VLDL and chylomicron and could be a marker of impaired HDL particle maturation. Additionally, the decrease in serotransferrin may lead to decreased protection against LDL oxidation.

Alwaili et al. used label-free quantitative proteomics based on spectral counting and the emPAI method to identify nine proteins, including hemoglobin subunit beta, ApoA4, serum amyloid A (SAA), haptoglobin-related protein (HRP), C3, gelsolin, carbonic anhydrase I, PGRP2, and fibronectin, with differential expression in acute coronary syndrome (ACS) patients [80]. The authors speculated that elevated SAA levels may account for improved cellular cholesterol efflux.

Weichhart et al. used label-free quantitative proteomics with the peptide index method to study HDL proteome in uremic patients. They determined that uremic HDL was enriched with surfactant protein B (SP-B), ApoC2, SAA, and *α*-1-microglobulin/bikunin precursor (AMBP) and demonstrated that SAA in uremia-HDL can promote inflammatory cytokine production [81].

Cubedo et al. analyzed serum and HDL samples from acute myocardial infraction (AMI) patients using 2DE and MALDI-TOF. They discovered that transthyretin (TTR; pI = 5.6, Mw = 42 kDa) decreased in patients with high cardiovascular risk [82]. Meanwhile, Huang et al. [6] also applied label-free quantitative proteomic approaches on HDLs in CAD patients and proposed clusterin reduction and ApoC3 increase as mechanisms leading to altered effects on endothelial apoptosis [82].

In summary, the changes in HDL protein expression detected by MS-based proteomic studies are observed in many types of ASVD or diseases with high ASVD risks. The alterations could manifest in apolipoproteins or other HDL-associated proteins, which compromise HDL lipid metabolism, antioxidation, anti-inflammation, antiapoptosis, immune regulation, or others functions. The changes in HDL protein quantity make HDL dysfunctional and lead to high ASVD risk.

## 7. Modification of HDL Lipoproteins as Potential Disease Markers

The quality of HDL is also an important marker for disease development. In nano-LC-MS/MS analysis, modifications and their locations on a protein can be identified. In MALDI-TOF-MS, it is beneficial to high throughput analyze apolipoprotein isoforms and obtain their relative abundance ratios. Therefore, in some cases, both techniques of nano-LC-MS/MS and MALDI-TOF-MS are applied to obtain complementary information. ApoC1 in HDL is a potent activator of LCAT and an inhibitor of CETP that can potentially regulate several lipase enzymes [83]. A functional polymorphism of ApoC1, T45S, was recently identified in some subjects of American Indian or Mexican ancestry [84]. More recently, a new full-length ApoC1_1_ (6721.6 Da) and its truncated isoform ApoC1_1_′ (6520.0 Da), each around 90 Da higher in mass than expected (ApoC1, 6631 Da, and ApoC1′, 6432 Da), were detected in a CAD cohort [85]. Oxidative ApoC1 and its oxidative-truncated form were specifically detected in HDL from patients with atherosclerotic vascular disease (ASVD), including CAD, carotid atherosclerosis, and ischemic stroke. Interestingly, there was no detectable oxidative ApoC1 in the plasma of these ASVD subjects, which may indicate that oxidative ApoC1 is specific to ASVD HDL. Therefore, oxidation of ApoC1 may be a useful marker for predicting CAD, carotid atherosclerosis, or stroke.

ApoA1 is an important activator of LCAT, and modified ApoA1 may compromise RCT and cause atherosclerosis [86]. Oxidation at Met112 of ApoA1 in HDL enhanced by the MxxY motif has been characterized as a sacrificial antioxidant protecting tyrosine from chlorination [87]. Myeloperoxidase- (MPO-) oxidized HDL may diminish the ability of ApoA1 to activate LCAT because oxidized ApoA1 Met148 disrupts the central loop overlapping the LCAT activation domain [88]. LCAT converts free cholesterol into cholesteryl esters, which are then sequestered into the HDL core for lipid metabolism. Lower LCAT activity could consequently aggravate cholesterol accumulation in arteries and lead to ASVD.

Therefore, oxidation at Met148 may be a more important factor than oxidation at Met112 in ApoA1 dysfunction. Using MALDI-TOF, we determined that high oxidation levels at Met112 are positively correlated with oxidation level of Met148* in vivo* [9]. Additionally, oxidation at Met112 and Met148 is higher in ASVD, uremia, and diabetes mellitus (DM) patients than in normal and primary hyperlipidemia (HP) groups, and oxidation at Met112 is highest in ASVD patients. Therefore, oxidation at Met112 and Met148 can increase risks of ASVD.

**Figure 5:**
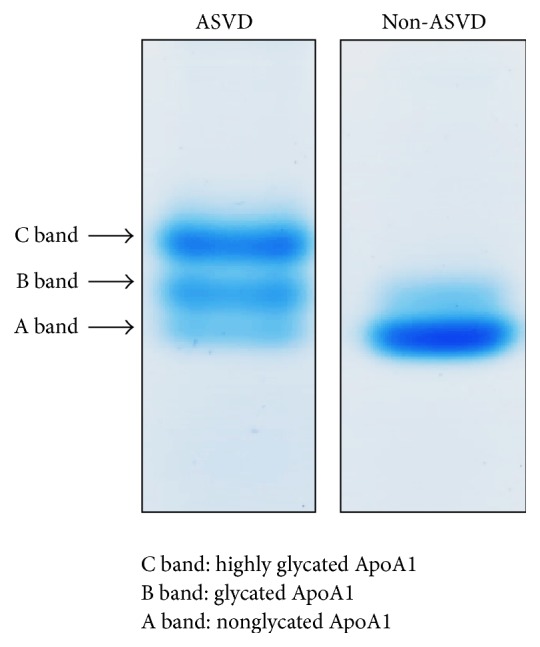
Bis-Tris (4%–12%) gel analysis of HDL samples. The blood was taken from one ASVD and one non-ASVD volunteer after getting informed consent, and the sampling protocol was approved by the institutional review board. Detailed experimental method can be referred to in [9].

ApoA1 can be glycated by covalent bonding of a sugar molecule. Glycation of HDL occurs in diabetes, uremia, and hyperglycemia [20, 89]. Glycated HDL is highly susceptible to oxidation, which induces endothelial cell injury and decreases atheroprotective effects against lipid peroxidation or oxLDL toxicity [90, 91]. Glycated ApoA1 can reduce RCT by decreasing ABCA-1 stability or by interfering with the contact between HDL and SR-B1 [92], a liver scavenger receptor that facilitates uptake of cholesteryl esters from HDL. Recently, glycation of ApoA1 was reported to impair its anti-inflammatory properties [93]. However, due to lower sensitivity of MS for negatively charged ions, glycated proteins/peptides are not easily detected. We recently used a gradient SDS gel (4–12%) to successfully separate glycated and nonglycated ApoA1 and found that higher levels of glycated ApoA1 specifically appear in ASVD patients (Figure 5). Subsequent purification of glycated ApoA1 allowed the weak signals of glycated peptides to be detected by MALDI-TOF.

ApoA1 in uremia patients has been reported to be heavily carbamylated due to the presence of high plasma urea levels [20, 94]. Urea degrades to cyanate and isocyanate, which exist in equilibrium. This electrophilic pair reacts with nucleophilic amino acids such as lysine in HDL proteins to induce protein carbamylation [95]. Lysine carbamylation (carbamyllysine) in ApoA1 can induce cholesterol accumulation in macrophages [20]. In addition to uremia, smoking is another cause of high plasma thiocyanate, which oxidizes to form cyanate and catalyzes ApoA1 carbamylation [94]. In addition to oxidation, glycation, and carbamylation, an increase in glycosylated ApoA1 levels was recently found in patients with AMI [96].

ApoC3 is present in three isoforms with 0–2 sialic acid molecules attached: ApoC3_0_, ApoC3_1_, and ApoC3_2_. ApoC3 kinetics was measured in an* in vivo* study, which suggested that all ApoC3 isoforms, especially the predominant C3_1_ and C3_2_ isoforms, contribute to hypertriglyceridemia. Additionally, ApoC3_2_ may be an important risk factor for cardiovascular disease because it has the most deleterious impact on LDL particle size [19]. Recently, the HDL-ApoC3/VLDL-ApoC3 ratio has been proposed as a potential predictor for CAD [45].

In summary, oxidation, carbamylation, glycation, or other modifications of apolipoproteins can compromise apolipoprotein and HDL-associated enzyme activities and result in RCT defect. The protein-modified HDL can thus lead to dyslipidemia and increase the hazard of ASVD.

## 8. Future Direction and Perspective

Due to the heterogeneity of HDL, the improvement of methods in HDL fractioning and purification is mandatory in the future. Despite MS being a well-developed technique, there are still pitfalls. Due to low MS sensitivity for ions carrying more negative charges, it is still hard to detect negatively charged modification of proteins and enzymes in the HDL protein mixtures. Enrichment of modified proteins or enzymes from HDL protein mixtures prior to MS analysis by stationary phase-coated materials or other purification methods (e.g., PAGE and LC chromatography) could be a more sensitive approach to identify modifications and its abundance. Recent development of target MS-based protein quantitation [97] can be an attractive method in the biomarker validation including their modifications in a large sample size of HDL.

## 9. Conclusion

Emerging development of MS proteomics provides a fast and sensitive analysis to discover markers or possible HDL roles in diseases. Lots of proteomic studies on HDL and subfractionation HDL have been reported and are mainly focused on atherosclerosis diseases. More recently, HDL protein modifications have been implicated as pathogenic factors directly or indirectly involved in atherosclerosis diseases.

Along with the tremendous technical progress in the field of MS-based proteomic studies, more sensitive and specific HDL modifications will be discovered and quantified. Using these HDL biomarkers, we will be able to more accurately predict the occurrence of ASVD.

## Figures and Tables

**Table 1:** Selected studies of MS-based HDL proteomics.

Disease	Study population	Quantitative strategy	MS approach	Validation	Major findings
CAD with PTCA [78]	CAD (*n* = 21)	^ 16^O/^18^O and iTRAQ labeling, IEF and SCX seperation	Nano-LC- MS/MS	None	Protective properties of HDL might be impaired after PTCA

ACS [98]	ACS (*n* = 40), healthy (*n* = 40)	2D-DIGE	MALDI-TOF-TOF	ELISA Western blot	Functional HDL subfractions shifted to dysfunctional HDL subfractions during ACS

CAD [99]	CAD (*n* = 6), ACS (*n* = 6), and healthy (*n* = 6)	1D-PAGE and label-free quantification (peptide index)	Nano-LC-MS/MS	ELISA IHC Western blot	A reduced clusterin and increased apolipoprotein C-III content of HDL in CAD and ACS as mechanisms leading to altered effects on endothelial apoptosis

Hemodialysis (HD) [79]	HD (*n* = 30), healthy (*n* = 30)	iTRAQ with IEF fractionation	LC-MALDI-TOF	ELISA	Increase of apoCII/apoCIII and the decrease of serotransferrin in HDL of HD patients

CAD ACS [80]	Healthy (*n* = 10), CAD (*n* = 10), and ACS (*n* = 10)	Label-free quantification (spectral counts and emPAI)	Nano-LC-MS/MS	Immunoblot or ELISA	Increased abundance of SAA, C3, and other inflammatory proteins in HDL from ACS patients suggests that HDL reflects a shift to an inflammatory profile

AMI [82]	AMI (*n* = 39), FH (*n* = 100), and healthy (*n* = 60)	2D-PAGE	MALDI-TOF	ELISA Western blot	TTR values are reduced in patients with high cardiovascular risk

Uremia [81]	ESRD (*n* = 24), healthy (*n* = 22), and CKD (*n* = 22)	1D-PAGE, label-free method with peptide index	Nano-LC-MS/MS	ELISA Western blot	SAA in ESRD-HDL can promote inflammatory cytokine production

CAD [8]	CAD (*n* = 18), healthy (*n* = 20)	MALDI-TOF peptide profiling	MALDI-TOF-TOF	MALDI-TOF	Developed a MALDI-TOF pattern containing peptides from apoA-I (oxidation at Met (112)), apoC-III (upregulated), lipoprotein(a) (upregulated), and apoC-I (downregulated) to classify CAD and control subjects

PTCA: percutaneous transluminal coronary angioplasty, ACS: acute coronary syndromes, AMI: acute myocardial infarction, CVD: cardiovascular disease, CAD: coronary artery disease, EBI: European Bioinformatic Institute, FH: familiar hypercholesterolemia, MI: myocardial infarction, MS: mass spectrometer, UC: ultracentrifugation, RA: rheumatoid arthritis, HD: hemodialysis.
